# Inhaled nitric oxide and acute kidney injury: new insights from observational data

**DOI:** 10.1186/s13054-017-1651-z

**Published:** 2017-03-31

**Authors:** Laveena Munshi, Neill K. J. Adhikari

**Affiliations:** 1 Interdepartmental Division of Critical Care, Sinai Health System, University of Toronto, Toronto, ON Canada; 2 Department of Critical Care Medicine and Interdepartmental Division of Critical Care, Sunnybrook Health Sciences Centre and University of Toronto, 2075 Bayview Avenue, Toronto, ON M4N 3M5 Canada

**Keywords:** Observational studies, Confounding, Propensity score, Nitric oxide, Acute kidney injury, Renal replacement therapy

The discovery of nitric oxide [1] generated excitement among intensivists because, as a therapeutic gas, it improved perfusion to ventilated lung units and increased arterial oxygenation without obvious systemic effects. Subsequent randomised trials in patients with acute respiratory distress syndrome (ARDS) found short-term improvements in oxygenation, with no effect on mortality and an unexpected increased risk of acute kidney injury (AKI) [2, 3]. In the absence of compelling biological mechanisms, one explanation could be that nitric oxide was used harmfully in trial protocols as opposed to clinician-directed practice. Although observational investigations can address this hypothesis, they are prone to bias and confounding that persist despite efforts at statistical ‘control’ during study design or analysis. However, empirical comparisons of treatment effects in randomised trials and observational studies have yielded mixed results [4, 5], and design alone does not determine the truth of study findings.

Ruan and colleagues recently published a retrospective cohort study (*n* = 547; 2007–2015) evaluating the relationship between inhaled nitric oxide administered in the first 3 days of ARDS and subsequent need for renal replacement therapy (RRT) [6]. They found that nitric oxide was associated with a substantial increase in RRT (adjusted hazard ratio 1.59, 95% confidence interval 1.08–2.34), consistent with meta-analyses of trials [2, 3]. The authors used propensity score matching to adjust for confounders, a stratified analysis to evaluate clinically important subgroups, and appropriate models to handle the competing risk of mortality. In a sensitivity analysis, residual confounding was evaluated using the rule-out approach, which concluded that an unmeasured binary confounding variable would have to be highly—and implausibly—associated with AKI and present much more frequently in the nitric oxide group to account for its observed nephrotoxicity. Notwithstanding the rigour of these analyses, we must consider their limitations.

The propensity score is the probability that a patient will receive a treatment, conditional on baseline characteristics entered into a logistic regression model with the treatment as the dependent variable. The score is used in several techniques to evaluate the effect of an intervention. One popular approach creates a group of patients who receive a treatment, each of whom is matched to at least one unexposed patient with the same propensity score. This method creates groups that appear to be well-balanced in a table of baseline characteristics. Unlike multivariable regression, propensity score-matched cohorts mimic the familiar structure of a randomised trial. Outcomes are compared directly between groups, preferably accounting for the matched nature of the data. However, propensity scoring does not overcome a fundamental limitation of observational studies: *only measured confounders can be assessed.* In addition, the ‘rule-out’ simulations to determine the necessary magnitude of a hypothetical confounder that would render the exposure harmless does not consider the more plausible scenario of several unmeasured confounders, each moderately associated with the outcome of AKI and more prevalent in the nitric oxide group. For example, it is possible that physicians who prescribed inhaled nitric oxide in the first 3 days of ARDS also preferred other more aggressive interventions, such as broader antimicrobials with more toxic side effects, or more frequent imaging with contrast media.

Another threat to study validity is that patients given nitric oxide may have had a worsening overall clinical trajectory prior to initiation, which cannot be modelled using baseline characteristics alone. If so, then patients who received nitric oxide may simply have been sicker, despite the similarity in propensity score, and thus more likely to receive RRT. Finally, concordant analyses of related outcomes, such as stage 3 AKI (which includes creatinine- and urine output-based criteria) and progression of AKI stage, would have reassured readers that the increased risk of RRT was not simply due to earlier physician initiation in these patients.

If we accept the finding that nitric oxide harms the kidney, what are the mechanisms? One reliable effect of nitric oxide is a short-term improvement in oxygenation. Recent studies have highlighted the increased mortality and organ failure associated with moderate and extreme arterial hyperoxia [7, 8], although the kidney may be spared [8]. Alternatively, or in addition, reactive nitrogen species formed as a product of nitric oxide and high fractional oxygen concentration delivery in ARDS may create a pro-inflammatory response leading to renal vasoconstriction and injury [3]. Given that these mechanisms are largely speculative, measurements of both nitric oxide metabolites and renal biomarkers in animals or patients with ARDS who receive this treatment are required to further elucidate the pathophysiology of AKI.

Like investigators using statistical tools to control confounding in observational datasets, intensivists have sought to control and correct physiologic abnormalities to improve patient outcomes. However, recent evidence in critical care has refuted the notion that normalized physiology benefits patients; examples include the survival advantage with lower hemoglobin, lower intensity ventilation, and higher blood sugar [9–11]. Similarly, interventions designed to moderate the intensity of ventilation to reduce ventilator-associated lung injury, such as prone positioning, pressure- and volume-limited ventilation strategies, and possibly extracorporeal support, have had greater impact on patient survival [9, 12, 13]. Given these findings, short-term physiological improvements are insufficient to justify an intervention in critically ill patients.

Health services researchers must settle for incomplete statistical control despite innovative and rigorous methods, while clinicians aim for physiologic understanding and sufficient rather than complete correction of abnormal parameters. Although the rule of rescue will likely mandate consideration of nitric oxide for patients with perceived life-threatening hypoxemia, the lack of survival benefit even in this dire setting [14] and increased risk of RRT should encourage transient use while more effective strategies are implemented. Since death from refractory hypoxemia in patients with ARDS is uncommon and nitric oxide is expensive, the resource-intensive side effect of RRT may simply reinforce efforts to curtail utilisation. Because additional trials of nitric oxide for ARDS are highly unlikely, future research on its renal effects should focus on mechanisms of AKI. In addition, a ‘big data’ approach would harness electronic patient record databases to explore whether temporal and hospital-level variation in nitric oxide utilisation for patients with ARDS is associated with AKI.

## Abbreviations

AKI: Acute kidney injury
ARDS: Acute respiratory distress syndrome
RRT: Renal replacement therapy
